# Editorial: Emotions in neuroscience: fundamentals and new discoveries

**DOI:** 10.3389/fnint.2024.1495554

**Published:** 2024-10-01

**Authors:** Giorgia Silani, Salvatore M. Aglioti, Daniela Perani

**Affiliations:** ^1^Department of Clinical and Health Psychology, University of Vienna, Vienna, Austria; ^2^Sapienza University of Rome and CLN2S@Sapienza, Italian Institute of Technology, Rome, Italy; ^3^Centre for Neurolinguistics and Psycholinguistics, Nuclear Medicine Unit, Vita-Salute San Raffaele University, Milan, Italy

**Keywords:** emotion perception, emotion expression, neuroscience, language, reward, fear, disgust, morality

This Research Topic collects articles from prestigious scientists partaking in the Emotions Itinerant Brain Forum, which addresses elements of emotional perception and expression from a biological, cognitive, and cultural perspective.

Starting from the expression and perception of basic emotions such as disgust and fear, Liuzza et al. propose a validated Italian version of the body odor disgust sensitivity scale—BODS, measuring individual differences in body odor disgust, a trait that plays an important role in understanding, for example, social behaviors.

Next, Frumento et al. analyze which perceptual features make an animal more-or-less scary to phobic and non-phobic people. By allowing participants to modify the spider's perceptual features (hairiness, body/leg size, and locomotion) in real-time on a computerized interface, their research aims to advance our knowledge of phobic preferences and improve the acceptability of exposure therapies.

Moving from individual to dual processing of emotions, Smekal et al. investigated the factors underlying naturalistic action recognition and understanding, as well as the errors occurring during recognition failures. In particular, they provide evidence on how form, motion, and temporal information differentially contribute to subjective action understanding in the context of naturalistic action perception.

In the language domain, Del Maschio et al. provide evidence that stronger emotional resonance underpins the processing of words in the native language of bilingual individuals, pointing to the different sensitivity of the hemodynamic responses to emotional information depending on the selected language.

Emotions also play a vital role at the social level, impacting various crucial aspects of work such as job satisfaction, performance, and employee wellbeing. In the mini-review from Boukarras et al., studies that have employed interpersonal (neuro)physiology to quantify the asymmetrical contagion of emotions in different contexts are examined. The review revealed that delayed synchronization of physiological states is a widespread phenomenon that may underpin the transmission of emotions, with implications for various aspects of organizational life, including leader-to-employee communication, and could drive the development of effective leadership training programs.

Understanding the neurobiology of reward processing is another important aspect of the realm of emotions. Two studies in this Research Topic address this topic. First, Bertrand et al. provide evidence of a diffuse limbic territory sensitive to reward within the subthalamic nucleus (STN) in non-human primates (macaca fascicularis). Then, Kraus et al. move to humans and systematically summarize available experimental results that assessed the modulation of social reward processing by intranasal oxytocin (IN-OXY) administration.

Finally, Sirgiovanni et al. investigate how complex social emotions such as shame and guilt differently impact the tendency to internalize the causality of negative events, attribute responsibility to themselves and others, and engage in responsible behavior. Findings indicate that guilt-prone people tend to attribute a higher degree of culpability to others, which is consistent with the view that guilt motivates people to choose the “moral paths in life.”

The overall Research Topic is a timely project as the study of emotions is not just important but necessary due to the significance of emotions in wellbeing and cultural change, which requires emotional intelligence from everyone to better understand each other as humans.

